# Detection of feline idiopathic cystitis as the cause of feline lower urinary tract disease in Sleman Regency, Indonesia

**DOI:** 10.14202/vetworld.2020.1108-1112

**Published:** 2020-06-16

**Authors:** Andi Tri Julyana Eka Astuty, Ida Tjahajati, Widagdo Sri Nugroho

**Affiliations:** 1 Graduate Program of Sain Veteriner, Faculty of Veterinary Medicine, Universitas Gadjah Mada, Yogyakarta, Indonesia; 2 Department of Internal Medicine, Faculty of Veterinary Medicine, Universitas Gadjah Mada, Yogyakarta, Indonesia; 3 Department of Veterinary Public Health, Faculty of Veterinary Medicine, Universitas Gadjah Mada, Yogyakarta, Indonesia

**Keywords:** cat, detection, feline idiopathic cystitis, feline lower urinary tract disease, Indonesia, Sleman

## Abstract

**Background and Aim:**

Feline lower urinary tract disease (FLUTD) is one of the common cat diseases. The aim of this study was to detect feline idiopathic cystitis (FIC) as a cause of FLUTD in Sleman Regency, which is a problem in the population.

**Materials and Methods:**

Seventy-three cats with FLUTD symptoms were used from seven veterinary practices in Sleman Regency. The logging of each cat’s medical history, clinical examination, urinalysis, routine blood screening, and ultrasonography was conducted to diagnose the cause of FLUTD.

**Results:**

The percentages of diseases causing FLUTD included FIC 21.9%, urolithiasis 57.5%, urinary tract infection (UTI) 16.4%, neoplasia 1.4%, trauma 1.4%, and nervous disorders 1.4%.

**Conclusion:**

FIC, one of the causes of FLUTD, is found in cats and has become a problem among the cat population in Sleman Regency, Yogyakarta, Indonesia. Various handling and preventive efforts should be undertaken against the disease.

## Introduction

Feline lower urinary tract disease (FLUTD) is one of the health disorders commonly found in veterinary practice. In general, the definition of FLUTD is a syndrome of various types of disorders occurring in the bladder and/or urethra of cats [1,2]. The clinical symptoms that can be found in the case of FLUTD include hematuria, pollakiuria, stranguria, periuria, dysuria, and either accompanied or not accompanied by an obstruction of the urethra [3,4]. Urolithiasis, urethral plugs, infections, neoplasia, anatomical defects, and iatrogenic reasons are some of the causes of FLUTD. However, if after an investigation does not find the cause of FLUTD, the feline patient is said to experience feline idiopathic cystitis (FIC) [2,5,6]. The diagnosis of FIC is made after eliminating other possible diagnoses in FLUTD [2,7].

In population studies conducted in Switzerland, 57% of FLUTD were caused by FIC, 22% by urolithiasis, 10% by a urethral plug, and 8% by urinary tract infection (UTI) [8]. Of the eight studies that have been done, the reported incidence of various causes of FLUTD, FIC is the main cause of FLUTD with a proportion between 27% and 72%; urethral plug occurs between 10% and 22%, UTI between 1% and 19%, and uroliths between 7% and 23% [6].

Information about FLUTD caused by FIC in Indonesia, especially in Yogyakarta, is still very limited. Veterinarians report encountering common symptoms of FLUTD, but a population-based depth review of this case has never been done. This investigation is the underlying research to examine FLUTD and FIC, further using the detect disease approach method in the population of cats in the Sleman Regency of Yogyakarta, Indonesia.

## Materials and Methods

### Ethical approval

All experimental protocols and animal work were approved by the Ethical Clearance Committee of the Veterinary Faculty, Gadjah Mada University, Indonesia (clearance number 0025/EC-FKH/Int./2019), and conducted in strict adherence to the principles of animal care.

### Study period and locations

The research was conducted from March to May 2019 in the following seven places in Sleman Regency: Animalova Small Animal Clinic, Klinik Hewan Jogja, Godean Pet Clinic, Satwa Kita Clinic and Pet Shop, Dji’o Pets Care, Kuningan Animal Clinic Faculty of Veterinary Medicine Universitas Gadjah Mada, and the Veterinary Hospital of Prof. Soeparwi.

### Procedures

This study used 73 cats (estimation of cats population data in Sleman Regency is 20,067 tails [9]) with symptoms of FLUTD (hematuria, pollakiuria, stranguria, periuria, dysuria, and both accompanied and not accompanied by obstruction of the urethra). The collection of medical history data, clinical examination, urinalysis, ultrasonography, and routine blood analysis was done to determine the cause of FLUTD.

The enforcement of urolithiasis and neoplasia diagnosis was performed if the ultrasound test results were known to be a stone/urolith or tumor mass [3,10]. The UTI diagnosis was made if the urinalysis observed concentrated urine with pyuria. Confirmation of the diagnosis of FLUTD disorders caused by trauma and paralysis was conducted based on the clinical examination of samples and the owner’s anamnesis. Verification of FIC was performed if the sample was not included in the group with urolithiasis, UTI, neoplasia, trauma, and paralysis [3,11].

### Statistical analysis

The data collected were analyzed descriptively using Microsoft Excel and SPSS 16.0 programs (IBM Corp., NY, USA).

## Results

### Sample variables

Domestic cats were 35.6%, Persian and Persian mixes were 27.4% and 31.5%, respectively. Male cats were found more than females. The average sample was 2.83±2.02 years old, with an average weight of 3.8±0.95 kg. When the entire sample was grouped by body condition score (BCS), cats with normal BCS and fat were greater than cats with lean BCS (Table-1).

**Table-1:** Description of cat samples in animal clinics in Sleman Regency.

Variable demographics	Frequency (tails)	Percentage (%)
Breed		
Domestic	26	35.6
Persian	20	27.4
Persian mix	23	31.5
Siamese mix	3	4.1
Angora mix	1	1.4
Gender		
Males	51	69.9
Females	22	30.1
Age		
<1 year	5	6.8
1-6 years	62	84.9
6-10 years	4	5.5
>10 years	2	2.7
BCS		
Lean (1-4)	3	4.1
Normal (5)	38	52.1
Obesity (6-9)	32	43.8

BCS=Body condition score

In this research, most cat owners complained of dysuria, hematuria, pollakiuria, and stranguria. Besides, 17.8% of samples came to the veterinary practice with non-specific general complaints, leading to urinary disorders (Table-2).

**Table-2:** The original anamnesis by the owner of the cat sample (in tails).

Diagnosis and number of patients (tails)	FIC (16)	Urolithiasis (42)	UTI (12)	Trauma (1)	Neoplasia (1)	Nerve disorder (1)	Total

Anamnesis							
Stranguria	4	7	2	-	1	-	14
Hematuria	6	10	2	1	-	-	19
Pollakiuria	8	8	1	-	1	-	18
Recurring	2	7	3	-	-	-	12
Dysuria	3	15	8	1	-	-	27
Others	1	11	-	-	-	1	13

FIC=Feline idiopathic cystitis, UTI=Urinary tract infection

### The proportion of causes of FLUTD

**Figure-1:**
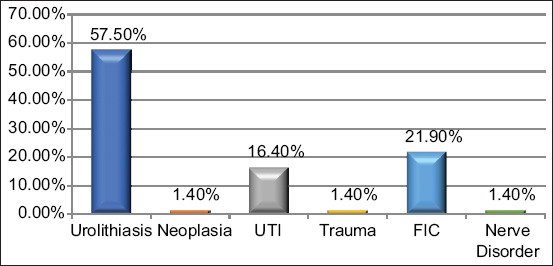
Diagnosis of feline lower urinary tract disease causes in cats in the Veterinary Hospital/animal clinic in Sleman Regency 2019.

Various causes of FLUTD were diagnosed in the 73 cats used, such as FIC found in 16 cases (21.9%), urolithiasis found in 42 cases (57.5%), UTI found in 12 cases (16.4%), neoplasia found in 1 case (1.4%), trauma found in 1 case (1.4%), and nerve disorders/paralysis found in 1 case (1.4%) (Figure-1). From these findings, it is known that FIC is found in the population of cats in Sleman district and is a problem in this population.

## Discussion

### Sample variables

Table-1 shows that domestic and Persian cats are the most common breeds of cats found in FLUTD research in Sleman district. The same was found in the previous studies, domestic and Persian cat breeds are more common as FLUTD cases than other breeds of cats [10,12]. Male cats comprised 69.9% of FLUTD cases, whereas female cats comprised 30.1%. In research on FLUTD conducted in Germany, the number of FLUTD cases in male cats was also more when compared with the incidence in female cats; this gender difference is significant in the case of FLUTD [3]. The anatomical difference between the urinary tract of male and female cats is one of the predisposing factors of males and might explain why males are more widely encountered when compared with females [10].

The overall age range of the sample is highly correlated between 0.5 and 10 years old, with an average sample age of 2.83 years. The average age of cats in the case of UTI is older when compared with other types of disease groups (Table-3). Similarly, earlier research mentioned that UTI is commonly encountered in older cats. The cat with UTI has a significantly older age when compared with a cat with FIC [3,7].

**Table-3:** Age, temperature, weight, and sex in each group of FLUTD causes.

Sample Demographic	FIC	Urolithiasis	UTI	Trauma	Neoplasia	Nerve disorder
Age (year)	2.5±1.53 (0.5-6.0)	2.76±1.63 (0.5-7.0)	3.93±3.34 (0.75-10)	0.5	1	2
Temperature (°C)	38.21±0.81 (36.1-39.5)	38.13±1.13 (33.6-39.7)	37.38±1.94 (32.4-39.3)	38.00	38.60	37.80
Weight (kg)	4.41±0.94 (3.0-6.5)	3.76±0.94 (1.0-6.0)	3.912±0.92 (2.8-6.0)	3.00	3.50	2.17
Gender (tails)	11 males, 5 females	28 males, 14 females	11 males, 1 female	1 male	1 female	1 female
Total (tails)	16	42	12	1	1	1

FLUTD=Feline lower urinary tract disease, FIC=Feline idiopathic cystitis, UTI=Urinary tract infection

The sample weight is in the range of 1-6.5 kg, with an average weight of 3.8±0.95 kg. The BCS assessment was conducted using a scale from 1 to 9. Sample grouping was done based on the BCS values of each sample. Samples are said to be lean/skinny if the BCS value is 1-4, normal body if the BCS is 5, and obese if the BCS is 6-9 [13]. After cats were grouped based on their BCS scores, there were more cats with normal and obese bodies and fewer cats with lean bodies. The average body temperature of the six groups of diseases indicated that they were in the ­normal range. This finding is in line with the previous studies that also found that the average body temperature in the case of FLUTD was 37.32°C±1.20°C [14].

In this study, cat owners provided very diverse medical histories of their cats with FLUTD symptoms. Some had only one complaint, but others came with more diverse and complex complaints. The owners’ complaints about each group of diseases were not very different from one another. This finding is in line with other studies that mentioned that based on the examination and medical history, reportedly, there is not much difference between diseases of FLUTD [3].

Pain during urination (stranguria) was a complaint in 14 cats. Nineteen cat owners provided bloody urine (hematuria) from their cats. Pollakiuria was observed in eight of 16 samples with FIC (50%) and was the highest proportion of the entire group of existing diseases that exist. A total of 17.8% (13 cats) of samples came into the veterinary clinic with unspecified general complaints, leading to urinary disorders, such as not eating and drinking, weakness, vomiting, and constipation.

Urinary disorders in medical history, such as stranguria, hematuria, pollakiuria, and dysuria, were commonly reported complaints encountered in the case of FLUTD [10,12,15]. Dysuria is difficulty in urination or the inability to urinate. It is commonly encountered in the case of FLUTD and is always accompanied by enlargement of the bladder. In this study, most owners complained that their young cat had difficulty or could not urinate due to the blockage of urine flow (obstruction). From the overall symptoms of FLUTD, obstruction is one of the symptoms that tend to be easily observed and is rarely ­overlooked by the owner. Cats with obstruction will generally become less active, have decreased appetites and drink less, look limp, and sometimes have vomiting. Cats with obstruction were significantly depressed and needed a veterinarian immediately [10].

### FLUTD events

After collecting medical histories, performing clinical examinations, and conducting supporting examinations, the cause of FLUTD symptoms was diagnosed. The diagnoses were FIC (21.9%), urolithiasis (57.5%), UTI (16.4%), neoplasia (1.4%), trauma (1.4%), and nerve disorders/paralysis (1.4%). A striking distinction was seen in the proportion of each disease in this study, as opposed to studies conducted in the Americas and Europe. In earlier research, FIC was the most common disease found as one of the causes of FLUTD, with the proportion of events above 50% [4,6,8,10,12,16].

The proportion of FIC (21.9%) in the FLUTD case in Sleman district is found to be similar to the results of research conducted in Thailand. The study reported that the proportion of FIC from 70 samples of cats with FLUTD symptoms was 27.1% [17]. Differences in the incidence of FLUTD cases in various studies can be due to several things, such as the characteristics of the population used in research, geographical location, animal signalement data, confounding factors, study approach method (prospective or retrospective), diagnostic criteria, inclusion factors, and sampling techniques of all research which can affect the outcome of the research conducted by each researcher [10,18].

Environmental factors are the basic concepts of epidemiology and play an important role in the disease process in the population. Environmental ­factors include biological, physical, and social events of the individual [19]. In this study, the intended purpose not only regarded the individual cat samples but also involved the environment of cats, which includes environmental factors, social, economic, and cultural owners who can influence the events of FLUTD. Differences in the proportion of cases of FLUTD in the cat population in Sleman, compared with other regions, can be due to differences in maintenance patterns, food management, sampling methods, knowledge levels, and cat owner awareness. Maintenance patterns, feeding management, the environment around cats, and the management of cat stress can affect the incidence of FLUTD [3,15].

The selection of food types for cats as pets is influenced by the decision of the owner; many clients have strong emotional ties with cats as their pets, they want the best for their cat health and survival. Good quality commercial cat food with balanced nutrients, good for digestion, meets the daily nutrition needs of cats, and safe for cats are important for owners [20]. Different things were learned in this research. Most cat owners are founder of food with a low price that is easy to obtain, regardless of the nutrient content, minerals, food quality, the number of gifts, needs of cats, and food safety.

This research used seven clinics/veterinary hospitals in Sleman Regency as an epidemiological unit. This sample may result in the measurement bias, caused by variations in the ability of the doctor in conducting a FLUTD diagnosis. In addition, not all veterinarians at each clinic/veterinary practice/veterinary hospital have the same interest in the field of urology. This difference can result in the occurrence of underdiagnoses in some instances. Similarly, a hypothesis was mentioned from research involving some clinical data in North America. The researchers added that the case of FLUTD was more commonly found in clinics with a particular interest in urology compared with other clinics, where the possibility of a case of FLUTD was not diagnosed as FLUTD in a clinic that had no particular interest in the field of urology [12].

The level of knowledge and public awareness of their cat’s health could be one of the reasons for the low detection of FIC in Sleman district. Cats with pollakiuria without obstruction are sometimes not observed by the owner or still considered normal because cats can still urinate. In some cases, cats still look active; therefore, for some owners, it is not yet considered a health issue that needs to be handled immediately by veterinarians. In FIC, the symptoms of FLUTD are seen to heal within 2-7 days by themselves, especially if not accompanied by an obstruction [5]. Unlike the urolithiasis cases observed in this research, the majority was followed by an obstruction (81%). When cats cannot urinate, abdominal pain arises, vomiting occurs, weakness follows, and depression results. This condition is evident and will be observed by the owner. It is deemed necessary to be handled by a veterinarian [10]. All these conditions can be reasons for the low number of FIC cases found in animal clinics in Sleman district. Besides, there are different characteristics of FLUTD itself in the cats population in Sleman district, compared with other research populations (America and Europe).

### Conclusion

From this research, it can be concluded that FIC is one of the causes of FLUTD that is found in the population of cats in Sleman, Indonesia, at a proportion of 21.9%. FIC is a problem in the population, and various handling and preventive efforts should be undertaken against the disease.

## Authors’ Contributions

IT, WSN, and ATJEA designed the study. ATJEA contributed to field survey and examined samples in the laboratory. All authors wrote, edited, read, and approved the final manuscript.
